# Attitudes, perceptions, and barriers of community pharmacists in Rwanda towards health promotion: a cross sectional study

**DOI:** 10.1186/s13690-022-00912-4

**Published:** 2022-06-23

**Authors:** Amon Nsengimana, Emmanuel Biracyaza, Jean Claude Hategekimana, Jacques Tuyishimire, John Nyiligira, Eugène Rutembesa

**Affiliations:** 1 Department of Pharmacy, School of Medicine and Pharmacy, University of Rwanda, Kigali, Rwanda; 2 Rwanda National Pharmacy Council (NPC), Kigali, Rwanda; 3 Programme of Sociotherapy, Prison Fellowship Rwanda (PFR), Kigali, Rwanda; 4 Department of Postgraduate studies, University of Kigali, Kigali, Rwanda; 5 Department of Clinical Psychology, School of Medicine and Pharmacy, University of Rwanda, Kigali, Rwanda

**Keywords:** Attitudes, Perceptions, Barriers, Community pharmacists, Health promotion

## Abstract

**Background:**

The practice of Pharmacists has changed worldwide over the past years. Today, health promotion is better known as an important part of modern pharmacy practice. Involving Community Pharmacists in health promotion is thus considered a valuable option in addressing public health issues. However, the literature on this practice remains unsubstantiated in African countries. In Rwanda, Community Pharmacists are believed to be solely involved in dispensing and very little has been studied about their role in health promotion. Thus, this study aimed to evaluate attitudes, perceptions, and barriers of Community Pharmacists in Rwanda towards their involvement in health promotion.

**Methods:**

A cross-sectional study was conducted among 236 licensed Community Pharmacists in Rwanda from 23rd January to 23rd June, 2021. A list of all respondents was obtained from Rwanda Food and Drugs Authority. All participants were randomly enrolled. Each community pharmacy was represented by one Pharmacist. We collected data from community pharmacy settings using a self-administered questionnaire made of close and open-ended questions. Statistical analyses were performed using Statistical Packages for Social Sciences (SPSS) version 25.

**Results:**

Of the 236 respondents, (*n* = 149, 63.1%) were male and (*n* = 87,37%) were female. The average age was 38.1 years (SD = 4.3). More than half confirmed that professional curriculum is adequate for offering health promotion services (*n* = 152, 64.4%).Majority responded that health promotion is part of their responsibility, and they are willing to provide health promotion services (*n* = 233,98.7%).The statement that “Pharmacists should not be involved in public health activities “was opposed by many (n=174,73.7%).The most sought-after service provided was education to drug misuse (n=211, 89.4%).Three major barriers to provision of health promotion were: lack of coordination with other healthcare professionals(n=106,69%),structure of healthcare system (n=157,67%),and lack of equipment (n=144,61%).Most Pharmacists disagreed with the statement that “patients are not interested in getting health promotion services”(*n* = 134,57%).

**Conclusion:**

Though Community Pharmacists faced several challenges that hindered their participation in health promotion, they had positive attitudes towards promoting public health messages. There are several barriers like lack of structure to provide health promotion services that need to be addressed to boost more active participation of Pharmacists in health promotion.

## Background

Health promotion refers to the process of enabling people to increase control over and improve their health [1]. This involves the population as a whole in the context of their everyday life, rather than focusing on people at risk for specific diseases [2]. As health promotion combines diverse, yet complementary methods, including communication, education, legislation, fiscal measures, organizational change, community development and spontaneous local activities against health hazards. It is a guiding concept involving activities intended to enhance individual and community health well-being [1–3]. This integral part of human life constitutes five key strategies for health promotion action in the charter and are: building healthy public policy, creating supporting environments, strengthening community actions, developing personal skills and reorienting health services [1, 4, 5]. Worldwide, pharmacy practice has changed and health promotion is currently known as an integral part of modern pharmacy practice [6, 7]. Although the literature on this practice in Sub-Sahara African (SSA) countries remains rare [8, 9], Community Pharmacists in countries like Ethiopia are known to play an integral role in promoting health [6, 10].

The joint International Pharmaceutical Federation (FIP) and World Health Organization (WHO) guidelines on good pharmacy practice incorporate health promotion as one of six components, which contribute to the health improvement of individuals that access community pharmacy [11]. Pharmacists are the third largest regulated healthcare professional group worldwide and community pharmacy is the most common discipline represented as an important contributor to the provision of health promotion activities to the individuals and society [12]. Community Pharmacists play a great role to optimize medication use and improve patient outcomes [13]. Pharmacists are now considered important members of health care teams involved in decisions about drug use and adverse effects, particularly in community and hospital pharmacies [14].

At the beginning of the twenty-first century, there were several calls to reform the pharmacy curricula to enable future Pharmacists to meet their new roles and responsibilities [15]. These new roles necessitate improving clinical knowledge and skills toward patients and communicating with patients and other health care workers [13, 16]. Pharmacists provide a critical service to society and interact with people from diverse backgrounds. Due to the easy access and friendly approach of Pharmacists, community pharmacies are among the first place patients visit to obtain consultation and treatment for their common ailments [17, 18]. Today, Community Pharmacists provide many public health services consisting of drug dispensing, medication therapy management, immunizations for children and adults, screening for diabetes, health education, consultation for a range of health risks and conditions such as diabetes, smoking cessation, weight management, hypertension, osteoporosis, substance abuse, verbal advice in response to symptoms and during the sale of over-the counter medicines and dissemination of general information [6, 8]. Despite these potential benefits of involving Pharmacists in public health activities, behavioural changes of both Pharmacists and public are needed. Pharmacists must accept their role in public health and make necessary changes in behavior to carry out the service [19, 20]. The public must also accept Pharmacists as providers of public health services and be willing to seek advice on some health issues from Pharmacists. To assist these behavioural changes, baseline studies characterizing the involvement of Community Pharmacists and the barriers hindering provision of health promotion service are very important [20–22].

While health promotion is basically an activity in the health and social fields, and not a medical service, health professionals, particularly in primary healthcare have an important role in nurturing and enabling health promotion. Health professionals should work towards developing their special contributions in education and health advocacy [1, 2]. Health promotion is a key element of public health and is applicable in the community, clinics, hospitals, and in all other health service settings [3]. Community pharmacies are considered an ideal site for credible counseling for a large segment of the population because Pharmacists are accessible, have frequent contacts with the public, have extended opening hours, are widely distributed geographically and absence of appointments needed for advice [20]. Community pharmacies hold a number of benefits as a setting for public health activities [7, 19]. This gives community pharmacies access to a range of individuals in both good and poor health, and to those that may not have contact with any other health professionals [19]. Many studies conducted elsewhere on the globe demonstrate that Pharmacist’s involvement in diabetes management resulted in reduced cost burden to the patients and improved overall treatment outcome and patient satisfaction [13, 18].

However, healthcare providers and Pharmacists in particular face a number of challenges to providing an efficient and effective health care services to the individuals and societies in which they serve for promoting health [22]. There have been several barriers that have hindered the provision of public healthcare services in community pharmacy settings such as: lack of knowledge and skills, confidence and adequate trainings and policies, poor recognition within the health care system, patients’ reluctance to use pharmacy services, and presence of inadequate number of pharmacy staff [23, 24]. In Ethiopia, lack of training, insufficient management support and lack of standard guidelines were identified as the top three barriers that limit the provision of health promotion services in community pharmacy [20].

Despite the barriers, Pharmacists are knowledgeable specialists who are currently under-utilized in the healthcare team [8]. There is a growing recognition that Pharmacists should be more involved in public health activities that fall within their scope and expertise due to accessible nature and frequent patient interactions [25, 26]. Adopting suitable health strategies to improve the involvement of Community Pharmacists in public health activities needs to be prioritized. Thus, the involvement of public health Pharmacists in the health care system has been expanding in the last decade. Countries like the United Kingdom, Pharmacists are integrated into public health programs and can play an important role in promoting health of individuals [20].

Community Pharmacists in Rwanda can as well play an important role to improve public health. Majority of Pharmacists in Rwanda work in community Pharmacies,only 39% work in the public sector [27]. This leads to an increased public access to community Pharmacies which may result in more clients seeking healthcare services in community pharmacies than in the public pharmacies. However, the level of willingness of Community Pharmacists in Rwanda to involve in public health services remains unstudied. This current study aimed to explore attitudes, perceptions, and barriers of Community Pharmacists in Rwanda towards provision of health promotion services. The findings will help healthcare system to increase utilization of the best practices found among Community Pharmacists and adopt strategies to improve their participation. Moreover, the findings will be a baseline for the healthcare system in Rwanda to revamp public-private partnerships and improve access to basic healthcare services in community pharmacies.

## Methods

### Study design

Across-sectional study design was conducted among Community Pharmacists in Rwanda from 23rd January to 23rd June, 2021. A validated self-administered questionnaire was employed in this study.

### Study settings and population

Rwanda is divided into four provinces (East, West, South, North) and the Capital city, Kigali. The provinces and the Kigali City are further divided into 30 districts [28]. The Pharmaceutical work force in Rwanda includes Pharmacists and Pharmacy technicians. Majority of Pharmacists work in the private sector, only 39% is serving in the public sector. Most Pharmacists in the private sector work in Kigali while 25% work in provinces [27]. Prior to practice of Pharmacy profession, Community Pharmacists are licensed by Rwanda National Pharmacy council (NPC). This study targeted all licensed Community Pharmacists in Rwanda.

This study was conducted in the registered non-individually and individually owned community pharmacies. All Community Pharmacists aged 21 and above with at least 12 months of the professional and working experience were included. We anticipated 21 years as the minimum age possible for a pharmacy graduate who has been working for at least 12 months. We excluded Community Pharmacists who have not worked for 12 months in Rwanda and who did not have a will to participate voluntarily in the study. Technically in Rwanda, community pharmacies are referred to as retail Pharmacies while Community Pharmacists are referred to as retail Pharmacists. Thus, in this study, we interchangeably used Community Pharmacists and community Pharmacies.

### Sample size and sampling

We used a list of licensed retail Pharmacies in Rwanda for the year 2021. On the list, Kigali city represented a greater number of retail Pharmacies with (*n* = 333, 57.6%) out of 578 [29]. We calculated the sample size of this study using the Yamane approach [8], $\mathrm{nY}=\frac{\mathrm{N}}{1+\mathrm{N}{\mathrm{e}}^2}$[30] where “N” stands for population size, and “e” for Alpha level (e = 0.05) at the confidence interval of 95%.Thus, $nY=\frac{578}{1+578{(0.05)}^2}=236$**.** Thereafter, we calculated sample from each province and the capital city, Kigali. We used stratum formula: np = (NP/N) *n Where: n = sample size, NP = number of pharmacies in each province or capital city before sampling, N = total number of all pharmacies in the targeted region and np = desired pharmacies in each region.

To recruit participants for this study, proportional stratified sampling was used to get the calculated sample of 236.Therefore, 136 of 333 Community Pharmacists were enrolled from Kigali city, 30 of 73 from the Eastern Province, 22 of 54 from the Western Province, 16 of 40 from the Northern Province and 32 of 78 from the Southern Province (Table 1).

**Table 1:** Number of the study participants by their location

City/Province	Kigali	Eastern	Western	Northern	Southern
Pharmacies	333	73	54	40	78
Sample	136	30	22	16	32

The number of participants were sampled using stratified proportion where majority of participants were selected from capital city, Kigali. This is because majority of the community pharmacies are found in capital city, Kigali

### Data collection

Data was collected by Pharmacists from 23rd January to 23rd June 2021.Prior to data collection, data collectors were trained on research ethics. A list of all licensed community pharmacies as of 2021 was obtained from Rwanda Food and Drugs Authority (Rwanda FDA). A total of 236 community pharmacies were selected randomly from this list. Each selected community pharmacy was represented by one Pharmacist. A self-administered questionnaire was distributed to Community Pharmacists in their working places (Community pharmacies). The research questionnaires were collected directly for minimizing the research bias that may happen during the study. The research questionnaire comprised of five main parts with close and open-ended questions. The first part asked 14 socio-demographics including: Sex, age, education level, marital status, religion, place of graduation, graduation year, location of pharmacy, pharmacy ownership, working experience in community Pharmacies, working hours per day, number of prescriptions to fill per day, age of most prevalent patients seeking healthcare services and availability of other health professionals. The second part explored attitudes and perceptions of Community Pharmacists towards provision of health promotion services, and we used Likert scale from (1 = strongly disagree to 5 = strongly agree). The third part inquired about different health promotion services provided by Community Pharmacists. We asked some questions like do you think that health promotion is part of Pharmacist’s responsibility, are you willing to provide health promotion services, if yes what you provide from the following: diabetes counseling, oral health, drug misuse, cardiovascular disease, nutrition, and physical activity. The fourth part examined the level of involvement of Community Pharmacists in provision of health promotion services. Eleven questions were used with Likert-scale (1 = very uninvolved to 5 very involved). The last part assessed barriers facing Community Pharmacists while providing health promotion services. We used Likert scale on 14 statements ranging from (1 = strongly disagree to 4 = strongly agree). The questionnaire we used was previously employed in other studies from Yemen and Malaysia [8, 13]. However, we did pre-test of this questionnaire to identify whether the items are reliable in Rwandan context. We conducted a pilot study among 20 Community Pharmacists and then adjusted the questionnaire for completeness. The results from pilot study were not included in the analysis. This instrument demonstrated a satisfactory internal consistency (Cronbach’s Alpha, α = 0.86).

### Data analysis

Upon completion of data collection, the responses from each participant were entered into the Statistical Package for Social Sciences (SPSS) version 25 for windows and double-checked for accuracy. The statistical analyses were performed using this software. The results were presented in text, tables, and charts. The first part of analysis was descriptive, and results were presented in frequencies, percentages, means, and standard deviation. The second part of attitudes toward the provision of the health promotion services, scores below the mid-point of 3 were taken as positive response or positive attitudes while those with greater than 3 points were considered as negative attitudes. The responses of disagree and strongly disagree on the subscale of attitudes of Community Pharmacists towards provision of health promotion services were combined and then calculated as the mean scores and standard deviation. The third part of health promotion services provided by Community Pharmacists, the scores above the mid-point of 3 were considered as positive response.

### Ethics

The authors assert that all procedures of this study comply with the ethical standards of the relevant national and institutional committee on human experimentation and with the Helsinki Declaration of 1975, as revised in 2008 [31]. The ethical clearance for conducting this study was requested and approved by Rwanda National Ethics Committee (RNEC), under reference number (No.1025/RNEC/2020). Prior to administering the questionnaire to the participants, explanation about the purpose of the study was given. The oral and written consent were obtained from participants who agreed to participate. Confidentiality was ensured during and after data collection.

## Results

The study results indicated that most of the respondents were male (*n* = 149, 63.1%) with overall average age of 38.1 ± 4.3 years. More than half of the respondents were single (*n* = 127, *n* = 53. 8%). A greater number of Community Pharmacists was in age group of 21-30 years (*n* = 138, 58.5%) followed by those aged 31-40 years (*n* = 88, 37. 3%). The education level of majority of Pharmacists was bachelor’s degree (BPharm) (*n* = 219, 92.8%). Regarding the place of graduation, majority of the respondents graduated from the University of Rwanda (*n* = 168, 71.2%). Most participants graduated between 2016 and 2020, (*n* = 143, 60.6%). The Religion of most of the respondents was Christianity, (*n* = 210, 89%). More than half of the respondents (*n* = 136, 57.6%) worked in the capital city, Kigali and (n = 168, 71.2%) were employees while (*n* = 68, 28.8%) were owners of the pharmacies. Of the study respondents, (*n* = 162, 68.6%) had less than 6 years of work experience in community pharmacy and majority (*n* = 108, 45.8%) worked 7-8 hours per day. Regarding the number of prescriptions to fill per day, more Pharmacists filled 1-20 prescriptions per day, (*n* = 83, 35.2%). Of the respondents, (*n* = 159, 67.4%) reported that most of their patients seeking health services were aged 18-49 years. Majority of the respondents indicated that other healthcare providers available in their community pharmacies were nurses, (*n* = 232, 98.3%) (Table 2).

**Table 2:** Demographic characteristics of Community Pharmacists and pharmacies

Variables	Frequency	Percent
**Sex**		
Male	149	63.1
Female	87	36.9
**Age**		
21-30 years	138	58.5
31-40 years	88	37.3
41-50 years	10	4.2
**Education**		
Bachelor’s degree	219	92.8
Master’s degree	12	5.1
Others	5	2.1
**Marital status**		
Single	127	53.8
Married	109	46.2
**Religion**		
Christian	210	89.0
Muslim	7	3.0
Others	3	1.3
Prefer not to say	16	6.8
**Place of graduation**		
University of Rwanda	168	71.2
Mount Kenya University	37	15.7
Foreign university	31	13.1
**Graduation year**		
> 2011	21	8.9
2011-2015	72	30.5
2016-2020	143	60.6
**Location of pharmacy**		
Kigali city	136	57.6
Southern Province	32	13.6
Northern Province	16	6.8
Western Province	22	9.3
Eastern Province	30	12.7
**Ownership of pharmacy**		
Owner	68	28.8
Employee	168	71.2
**Professional experience in community pharmacy**		
1-5 years	162	68.6
6-10 years	58	24.6
11-15 years	9	3.8
16-20 years	5	2.1
More than 20 years	2	0.8
**Working daily hours**		
< 7 hours	6	2.5
7-8 hours	108	45.8
9-10 hours	77	32.6
11-12 hours	25	10.6
> 12 hours	20	8.5
**Number of prescriptions per day**		
1-20 prescriptions	83	35.2
21-40 prescriptions	69	29.2
41-60 prescriptions	31	13.1
61-80 prescriptions	12	5.1
81 and above prescriptions	17	7.2
Not sure	15	6.4
Prefer not to say	9	3.8
**Age of most prevalent patients seeking service**		
< 18 years	18	7.6
18-49 years	159	67.4
50 and above	10	4.2
Not sure	40	16.9
Prefer not to say	9	3.8
**Availability of other health professionals in the pharmacy**		
Nurse	232	98.3
Nutritionist	1	0.4
Others	1	0.4
Only Pharmacist	2	0.8

### Involvement of community pharmacists in health promotion activities

**Fig. 1:**
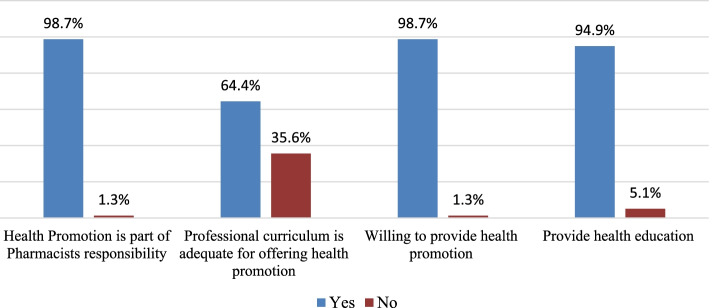
Willingness of community pharmacists to the involvement in health promotion activities

More Community Pharmacists, (*n* = 233, 98.7%) responded that health promotion is part of their responsibility. Majority showed that professional curriculum is adequate for offering health promotion services, (*n* = 152, 64.4%). Regarding the will to provide healthcare services, (*n* = 233, 98.7%) showed that they had a will to provide health promotion services as it is their professional responsibility. Of the respondents, (*n* = 244, 94.9%) provided health education and promotion programs in their pharmacies (Fig. 1). Our results indicated that the involvement of the respondents in healthcare provision is at a good level. Of the respondents,(*n* = 125,53%) indicated that they were involved in providing asthma counseling,(*n* = 118,50%):health education for diabetic patients,(*n* = 117,49.6%):health education to patients with cardiovascular diseases (CVD),(*n* = 119,50.4%):nutrition and physical activity counselling,(*n* = 102,43.2%):special population counseling,(*n* = 91,38.6):education on smoking cessation,(*n* = 127,54%):oral health counselling,(*n* = 111,47%):weight management counseling,(*n* = 75,31.8%):immunization counseling, n = (*n* = 40,17%):cancer counseling. Majority indicated they were highly involved in providing education to drug misuse (*n* = 121,51.3%) (Table 3).

**Table 3:** Involvement of Community Pharmacists in health promotion activities

Level of involvement	Asthma counseling	Diabetes counseling	CVD counseling	Drug misuse	Special population counseling	Nutrition and Physical activity counseling	Smoking cessation counseling	Oral health counselling	Immunization counseling	Traditional and complementary medicine counseling	Weight management counseling	Cancer counseling	FP counseling
Very uninvolved	13	8	8	7	10	8	12	10	25	15	11	42	37
Uninvolved	14	6	6	6	12	20	22	14	36	40	17	51	49
Uncertain	56	38	38	17	46	46	74	44	89	80	57	71	78
Involved	125	118	117	85	102	119	91	127	85	71	111	40	53
Very involved	28	65	65	121	66	43	37	41	11	30	40	32	19

*FP* Family planning, *CVD* Cardiovascular diseases

Concerning involvement of Community Pharmacists in delivering health services, the participants expressed that they play a role in health promotion and disease prevention to increase the quality of life of their clients seeking healthcare services. Community Pharmacists openly expressed how they see provision of health promotion services.

“*The practice of health promotion is emerging with the fact that the number of health promoters are rising probably due to the educational promotion. As Rwanda is developing, diseases related to the development are also increasing. In short, health professionals especially Community Pharmacists and nurses have a huge task to deliver health promotion advice*.”

“*Community Pharmacists used to focus on drug dispensing but for the moment many people are aware that Pharmacists are also there for health promotion services. In many circumstances the clients come to ask for health information in community pharmacy as they don’t like to seek services or health-related information in the hospitals*.”

### Type of health promotion activities provided by community pharmacists

Community Pharmacists enrolled in this study showed that they provided several healthcare services in their daily work. The most sought-after service that they delivered was education to drug misuse, 89.4% (*n* = 211). Regarding sexual and reproductive health, 84.7% (*n* = 200) delivered emergency oral contraception and 78.4% (*n* = 185) provided counseling to partners initiating treatment for sexually transmitted diseases. Among non-communicable diseases (NCDs), diabetes counseling was the most provided 83.5% (*n* = 197), followed by counseling on cardiovascular disease (CVD) 67.8% (*n* = 160). Of the respondents, 69.5% (*n* = 164) provided oral health counseling. Psychological counseling was provided by 84.7% (*n* = 200) of the Pharmacists. About providing education on risk factors for chronic disease, 55.5% (*n* = 136) of Community Pharmacists provided counselling on weight management while 74.6% (*n* = 176) provided counselling on nutrition and physical activities. Of the respondents, 55.5% (*n* = 131) provided asthma counseling and 77.1% (*n* = 182) provided traditional and complementary medicine counseling. The three least provided health promotion services were: counseling special population (Young, old, pregnant, and immune compromised people):42.8% (*n* = 101), smoking cessation:53.4% (*n* = 126) and immunization counseling:53.8% (*n* = 127) (Table 4).

**Table 4:** Different public health activities provided by Community Pharmacists

Health promotion activities	Yes		No	
	Number	Percent	Number	Percent
Diabetes counseling	197	83.5	39	16.5
Oral health counseling	164	69.5	72	30.5
Drug misuse counseling	211	89.4	25	10.6
Cardiovascular disease counseling	160	67.8	76	32.2
Nutrition and physical activity counselling	176	74.6	60	25.4
Smoking cessation counselling	126	53.4	110	46.6
Psychological counseling	200	84.7	36	15.3
Asthma counseling	131	55.5	105	44.5
Immunization counselling	127	53.8	109	46.2
Weight management	136	57.6	100	42.4
Counseling special population (YOPI)	101	42.8	135	57.2
Traditional and complementary medicine counseling	182	77.1	54	22.9
Emergency oral contraception	200	84.7	36	15.3
Counseling partners when initiating treatment for sexually transmitted diseases	185	78.4	51	21.6

### Barriers to the involvement of community pharmacists in health promotion activities

**Fig. 2:**
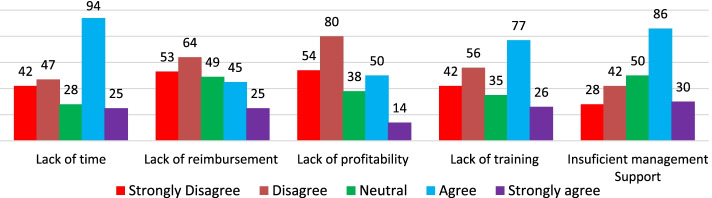
Barriers to the involvement of Community Pharmacists in public health activities (part 1)

**Fig. 3:**
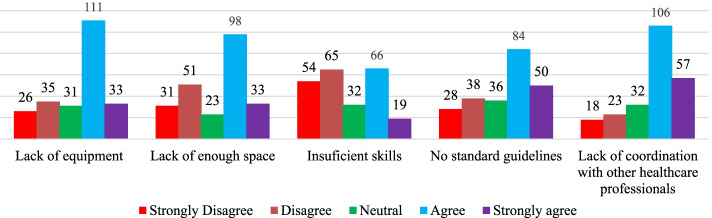
Barriers to the involvement of Community Pharmacists in public health activities (part 2)

**Fig. 4:**
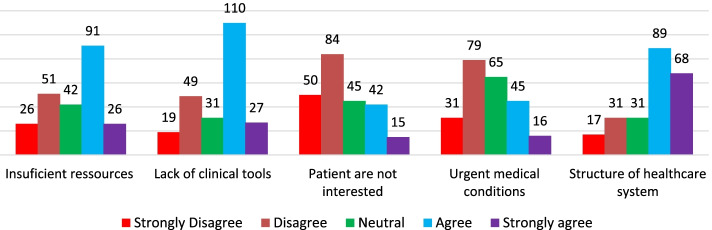
Barriers to the involvement of Community Pharmacists in public health activities (part 3)

Regarding the barriers to provision of healthcare services, majority responded that they encounter several barriers. Among the barriers, majority cited lack of coordination with other healthcare professionals, structure of healthcare system and lack of equipment as the major barriers (Fig. 2). The respondents also showed that lack of time, lack of enough space, lack of skills enough to provide service,no standard guidelines available for offering the service, insufficient trainings or lack of trainings and insufficient management support were the barriers to promoting health of clients seeking health services in community pharmacies (Fig. 3). Though majority of the participants agreed that lack of personnel/resources and lack of clinical were among barriers, majority disagreed with the barrier that patients are not interested or has more urgent medical conditions (Fig. 4).

Community Pharmacists openly expressed several perceived barriers affecting public health practices in community pharmacies and these barriers also hindered their role in promoting health”.

“We, Community Pharmacists*, are somehow limited by some health policies governing pharmacy profession in Rwanda. So far so good but we are still progressing because there are so many other health services that Community Pharmacists are not allowed to perform*.

Community Pharmacists expressed that the regulatory authorities do not allow provision of some healthcare services in community Pharmacies, yet these services would contribute to health promotion. “*They suggested services like diabetes screening, immunization, contraceptives injection, vaccination, laboratory test like Malaria test, HIV test and pregnancy test,*”

“*We, Pharmacists, face challenges such as lack of trainings, lack of structure to provide services beyond dispensing, and lack of standards guidelines to provide health promotion services*.”

“*The socio-cultural barrier is one of the barriers to provision of health promotion services. There are clients who think that Pharmacists are only specialized in drugs. Some have perceptions that pharmacies are like other shops with the aim of getting money. Thus, some clients wish to be treated based on their needs instead of following our recommendations or Doctors’ prescription*.”

“*We face lack of supporting documents on health promotion, insufficient knowledge on extended healthcare services, lack of equipment dedicated for the patients such as BP Monitoring machine, weighing scale, limited time for providing services and lack of structure on other health services*.”

“*Some pharmacy owners are money oriented, and it limits Pharmacists to keep professionalism, Pharmacists have limited time, conflict of interest with other healthcare professionals and lack of training. These also hamper the quality of health services we must provide*.”

“*Nowadays, it is backward not only due to COVID-19 but due to the presence of some business-oriented Pharmacy Owners. Some owners, their sole purpose is income or monetary means, they don’t focus on the wellbeing of patients*.”

“*Health promotion in community Pharmacy is a little bit scarce as the most important thing clients want in pharmacy is essential medicines to their medical complaints and some pharmacies want most of the time making profit in this service*.”

Other Community Pharmacists stated that lack of trainings, lack of enough space to provide health promotion services, inadequate cooperation with their clients and staff, lack of standard guidelines and socio-cultural influences hindered their involvement and provision of health promotion services:

### Community pharmacists’ attitudes towards public health activities

On average, majority of Community Pharmacists who were enrolled in the current study possessed positive responses to the statements about their attitudes towards public health activities. Our results indicated that majority of the respondents reported positive statements or attitudes while providing healthcare services (Table 5).

**Table 5:** Attitudes of Community Pharmacists

Statements	M (SD)
Pharmacists should not be involved in public health activities	1.6 (1.2)
People will not accept my participation in public health activities	1.7 (0.9)
I am not ready to be involved with public health activities.	1.5 (0.8)
It is not important for Pharmacists to practice health promotion activities.	1.4 (0.8)
Health education only to problems related to drugs should be provided.	2.1 (1.1)
I am not interested in public health activities as it is the work of doctors and nurses	1.4 (0.7)
I don’t have enough knowledge to advice patients on health promotion and disease prevention.	1.5 (0.8)
Public health activities belong to health centers	1.6 (0.8)
I do not have the time to educate patients on health issues.	1.7 (0.9)
Other health workers do not allow Pharmacists to carry out public health activities.	2.6 (1.2)

In Table 6, our results indicated that majority of the participants had positive attitudes. Majority indicated that they strongly disagreed with items such as “Pharmacists should not be involved in public health activities” (*n* = 174, 73.7%), “people will not accept my participation in public health activities” (*n* = 119, 50.4%), “I am not ready to be involved in public health activities” (*n* = 161, 68.2%), “it is not important for Pharmacists to practice health promotion activities” (*n* = 168, 71.2%), “I am not interested in public health activities as it is the work of doctors and nurses” (*n* = 170, 72%), “I don’t have enough knowledge to advice patients on health promotion and disease prevention” (*n* = 159, 67.4%), “public health activities belong to health centers” (*n* = 132, 55.9%), “I do not have the time to educate patients on health issues” (*n* = 124, 52.5%). Respondents dominantly disagreed that health education only to problems related to drugs should be provided (*n* = 95, 40.3%). They slightly disagreed with the statement that other health workers do not allow Pharmacists to carry out activities related to public health (*n* = 63, 26.7%) (Table 6).

**Table 6:** Community Pharmacists ‘attitudes towards provision of public health activities

Attitudes of respondents	Strongly disagree		Disagree		Neutral		Agree		Strongly agree	
	N	%	N	%	N	%	N	%	N	%
Pharmacists should not be involved in public health activities	174	73.7	36	15.3	0	0	7	3	19	8.1
People will not accept my participation in public health activities	119	50.4	90	38.1	17	7.2	6	2.5	4	1.7
I am not ready to be involved with public health activities	161	68.2	56	23.7	3	1.3	5	2.1	5	2.1
It is not important for Pharmacists to practice health promotion activities	168	71.2	55	23.3	5	2.1	4	1.7	4	1.7
Health education only to problems related to drugs should be provided	83	35.2	95	40.3	23	9.7	26	11	9	3.8
I am not interested in public health activities as it is the work of doctors and nurses	170	72	59	25	1	0.4	2	0.8	4	1.7
I don’t have enough knowledge to advice patients on health promotion and disease prevention	159	67.4	65	27.5	4	1.7	5	2.1	3	1.3
Public health activities belong to health centers	132	55.9	80	33.9	12	5.1	9	3.8	3	1.3
I do not have the time to educate patients on health issues	124	52.5	80	33.9	20	8.5	8	3.4	4	1.7
Other health workers do not allow Pharmacists to carry out activities related to public health.	57	24.2	63	26.7	57	24.2	44	18.6	15	6.4

N: Frequency

## Discussion

This study evaluated attitudes and practices of Community Pharmacists in Rwanda to promote health through active participation in public health activities and the barriers preventing their participation. Consensus was seen with the preceding studies conducted among Community Pharmacists [32], as our results revealed that Community Pharmacists in Rwanda had positive attitudes to involve in different public health services that lessen public-health problems among patients seeking healthcare services in community pharmacies. Our results revealed that Community Pharmacists were involved in health educational and counseling activities related to personal lifestyle practices. The results revealed that almost all Community Pharmacists witnessed that patients seeking healthcare services accept their participation in public health activities and thus Pharmacists were ready to be involved in these indispensable activities for health promotion. Our results are parallel to the previous studies that conveyed how Community Pharmacists can play an important role in public health and they need to be competent in different areas of practice [13, 33]. There are three domains that Community Pharmacists can be involved in: health protection and prevention, health and social care, and health improvement [18, 34]. The feedback obtained from most Community Pharmacists showed positive attitude towards involvement in public health activities and recognition of its vital contribution. Our results are in line with the preceding studies that documented positive attitude of Community Pharmacists towards public health practices even though the knowledge and practice level were not satisfactory [10, 12, 13, 32].

Consistent with the previous studies including a systematic review that indicated the crucial role of Community Pharmacists in providing healthcare services and the barriers to participating in public health activities [19, 35], our findings discovered that several Pharmacists had will to be part of public health activities, they had interests in supporting public health activities as part of their healthcare team and had experiences that support them in preventing diseases for health promotion. Our study indicated a high level of participation of Community Pharmacists in public health activities in Rwanda. These findings are similar to prior studies [13, 36, 37], however they are in contrast to studies conducted in Ethiopia and Nigeria that indicated a low level of participation of Community Pharmacists in public health activities [26]. In this study, fourth-fifth of respondents claimed that other health workers do not allow Pharmacists to carry out public health activities. Hence, good communication and cooperation between different health care professionals should be established [8, 26]. However, the public acceptance of participation of Community Pharmacists in public health activities was perceived positively. All these findings suggest that there is an acceptance from both sides of Community Pharmacists and patients towards changing from the traditional role of dispensing medication to more effective involvement in health care activities.

Community Pharmacists in Rwanda felt it was important for Pharmacists to practice health promotion activities. They also had a positive opinion regarding providing health education beyond drug-related issues and believed that public health activities are not just the responsibility of doctors and nurses. Our results are similar to prior studies [13, 36]. The current findings showed that Community Pharmacists have competent knowledge to provide counseling on health promotion and diseases prevention to patients who visit community pharmacies seeking healthcare services. The public health activities they provide include oral health counseling, education on risk factors of non-communicable diseases (NCDs) including health nutrition, physical activities, education on weight management and cholesterol balance, education on smoking cessation that may decimate their quality of life. They also provide health education on cancer, immunization, drug misuse, family planning and immunocompromised patients. Our results correlate with prior studies that found that Community Pharmacists have great contributions to the provision of wide range of public health services such as health education, prevention of diseases, provision of medications, counseling on rational use of medicines,educational consultations, medication management and other medication optimization services, chronic condition management, patient empowerment, public healthcare coordination, health and wellness services of the patients [13, 36–38].

In agreement with previous studies that conveyed that Community Pharmacists encounter several barriers such as lack of reimbursement, time and training strains, insufficient time for healthcare provision, insufficient management support, unavailability of guidelines to provide services and lack of profitability [13, 32], our results revealed that Community Pharmacists in Rwanda experienced a number of barriers hindering their involvement in promoting health of patients seeking health services in community pharmacies. Among those barriers, there were: structure of healthcare system, lack of equipment, lack of time, understanding of the concept of teamwork among different healthcare professionals, poor conceptualization of public health, insufficient training, poor recognition from their clients, insufficiency of specialists and professional experiences, lack of official recognition of participation in public health activities. Our results agree with previous studies conducted in SSA countries such as Ethiopia and Nigeria [13, 32, 36]. The reported barriers can be partly overcome if there is an inclination to deliver unpaid healthcare services rather than charging services to improve business profitability by Community Pharmacists. The concept of inter-professional collaborative teamwork must also be embraced between Community Pharmacists and medical Doctors. This multidisciplinary work may play a great role in promoting health of patients seeking healthcare services from community Pharmacies. Our results are similar to prior findings that documented key areas including inter-professional collaborative care as initial steps for the involvement of community pharmacies in public health activities [39].

### Strengths and limitations

There were a number of strengths and limitations within this study. Firstly, the present study used validated data collection instrument that was previously used in similar studies. This instrument indicated a satisfactory consistency. Secondly, this study was the first research conducted at a national level among Community Pharmacists to investigate the level of Community Pharmacists in promoting health through providing public health practices to clients seeking healthcare. Random Sampling technique was applied which included Community Pharmacists from all regions of the country (East, West, South, North and the capital city). This allowed us to generalize at a national level the level of involvement of Community Pharmacists in Rwanda towards health promotion services as well as barriers they encounter. Thirdly, the use of mixed methodology within this study helped to ensure consistency and validity of our results and obtain a better understanding of the phenomenon based on the perspectives from the respondents. However, we encountered a few limitations during this study. One of which related to the limited and insufficient literature on Community Pharmacists in SSA and Rwanda specifically. Second to this, relying on self-reported information from Community Pharmacists and conducting the study in the period of Coronavirus pandemic (COVID-19) might also contribute to bias in outcomes.

## Conclusions

To conclude, community pharmacists had positive attitudes as far as providing health services is concerned; however, they face some challenges that need to be addressed for increasing the level of their participation in public health activities and promoting quality of health of the patients seeking health services from community pharmacies. Our results proved and implied that community pharmacists in Rwanda are involved in health promotion practices and they have positive attitudes towards public health activities. Furthermore, these activities are provided at a competent level despite the barriers encountered. The major barriers that prevented their effective contribution as public health practitioners include profitability strains, insufficient management support, lack of time and lack training, no standard guidelines, and lack of official recognition for their involvement in providing services. There is a need to improve and clarify structure of provision of healthcare services in community pharmacies which are close to the community and lead by highly trained pharmacists. For strengthening the contribution of community pharmacists on public health activities that fall in their expertise, a further study on evaluation of the contribution of community pharmacists and factors associated with their involvement and quality of their services are essential in Rwanda. Community pharmacists must be taken as important members of the health care team and policymakers are recommended to design appropriate procedures aiming to improve the involvement of pharmacists in providing health services in Rwanda.

## Data Availability

All relevant data are included in this manuscript. Data may be shared upon reasonable request is provided to the corresponding author.

## Abbreviations

FIP: International Pharmaceutical Federation
YOPI: Young, Old, Pregnant women, Immune-compromised
RNEC: Rwanda National Ethics committee
NPC: National Pharmacy Council
